# Analysis of the Possibility and Conditions of Application of Methylene Blue to Determine the Activity of Radicals in Model System with Preaccelerated Cross-Linking of Polyester Resins

**DOI:** 10.1155/2019/2879869

**Published:** 2019-08-01

**Authors:** Karol Hęclik, Jerzy Duliban, Barbara Dębska, Jacek Lubczak

**Affiliations:** ^1^Rzeszow University of Technology, Faculty of Chemistry, Department of Biotechnology and Bioinformatics, Al. Powstancow Warszawy 6, 35-959 Rzeszow, Poland; ^2^Rzeszow University of Technology, Faculty of Chemistry, Department of Organic Chemistry, Al. Powstancow Warszawy 6, 35-959 Rzeszow, Poland

## Abstract

Unsaturated polyester resins are usually processed using a curing system consisting of initiator and accelerator introduced into the resin. Actually, the producers apply built-in amine accelerators which can be named as preaccelerators. Commonly used preaccelerators for unsaturated resins are tertiary aromatic amines of which incorporation into resin structure may bring better stability. It also causes shorter gelation time of resins because of formation of active RO• radicals that initiate polymerization. Investigated radical reactions are too fast and there is no possibility of freezing it (in unsaturated polyester) to measure with Electron Paramagnetic Resonance (EPR). The analytical methodology on radicals activity measurement in model of preaccelerated unsaturated polyester resin reaction with methylene blue as indicator was presented. Using methylene blue as indicator allows us to determine the activity of forming radicals in three-component system (cobalt salt, amine preaccelerator, peroxide, or hydroperoxide) during the reaction of radicals generating. Changes in radicals activity using methylene blue as interceptor can be observed by changes of transmittance in the UV-Vis spectrum in the range 400-950 nm.

## 1. Introduction

Unsaturated polyester resins polymerization reactions are initiated by using a suitable initiator; the most commonly used are organic peroxides or hydroperoxides of aliphatic ketones. These substances initiate very fast polymerization reactions at room temperature (25°C). To further accelerate the polymerization process and shorten the gelation time of unsaturated polyester resins, additional modifiers (called preaccelerators or polymerization accelerators) in the form of aromatic amines are used. Such systems, where peroxide and accelerator are built-in, have reducing properties and are called redox systems. The presence of accelerating substances has a selective effect on aliphatic ketone peroxides and results in a reduction of the decomposition energy of peroxide bonds. The system containing peroxide and amine accelerator is also a redox system. On the other hand, the additional presence of accelerators selectively affects the aliphatic ketone peroxides, resulting in the reduction of the decomposition energy of peroxide bonds. The use of salts of metal such as vanadium, manganese, or cobalt results in the decomposition of the peroxide or hydroperoxide of aliphatic ketones and also the radical formation process is accelerated. Cobalt compounds in the form of cobalt(II) octoate are the most commonly used salts due to the fact that the curing process can be carried out over a wide range of temperatures: 20-100°C [1]. The use of aromatic amines in the curing process of unsaturated polyester resins is of great importance as it significantly increases the gelation rate of such modified polyester resins. Unsaturated polyester resins with benzoyl peroxide as a curing system are already used at room temperature [2]. However, ternary systems consisting of unsaturated polyester resin dissolved in styrene as a comonomer with a peroxide initiator and addition of an aromatic tertiary amine are usually used in industrial practice [3–9]. These compositions are very reactive even at temperatures as low as 5°C [1]. Modifiers with aromatic or heterocyclic rings embedded with various substituents with a greater number of hydroxylamino functional groups (capable of reacting with components used for the synthesis of unsaturated polyester resins) are also used [10–14].

The effects of amine as a promoter of cobalt-curing are described [15, 16] both when amine was built into polyester chain structure, and when it creates a physical mixture with unsaturated polyester resin but is not chemically bound:

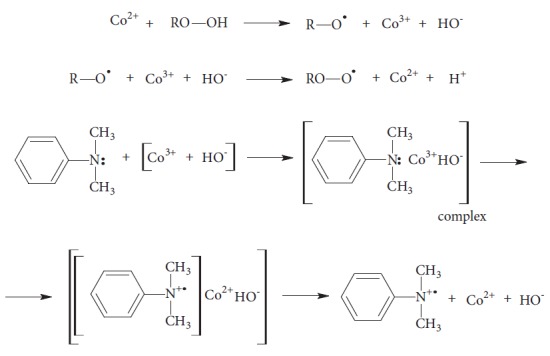
(1)


The mechanism shown above allows explaining the phenomenon of a reduction in gelation time of unsaturated polyester resin modified with tertiary aromatic amines incorporated in polyester chain or mixed with unsaturated polyester resin after prior inserting cobalt(II) salt. It is effect of phenomenon of Co^3+^ ions complexation in first step which are created in reactions with cobalt Co^2+^ salt and hydroperoxide. The next step involves decomposition of created coordination complex and composition of aromatic cation radical which react with double bonds located in polyester chain or with styrene double bonds. These processes lead to formation of active radicals RO• which initiate the cross-linking of the resin. Amines can also lead to hydroperoxide disintegration and thus to cure the resin [15, 16].

Radicals are highly reactive uncharged molecules or atoms that arise as a result of unpaired valence electrons. They can be detected using the technique known as EPR. There are no restrictions to the physical state of a sample: gas, liquid, or solid state is acceptable. Limitations are due to very short lifetime of radicals: less than 10^−6^ s which conduces to the need of freezing samples. However, there are reactions that cannot be extremely fast frozen or reactions in which changes of radicals' activity need to be measured during the reaction path. Investigated tertiary aromatic amines in reaction with peroxides give radicals, which causes subsequent reactions, and it is important to observe those changes. Adding to the system a methylene blue as indicator allows observing this process by transmittance value changes, for the wavelength of electromagnetic radiation in the UV-Vis spectra range. As a result of the redox process, the colour of the indicator changes from blue to colourless (leuco form) [17]:

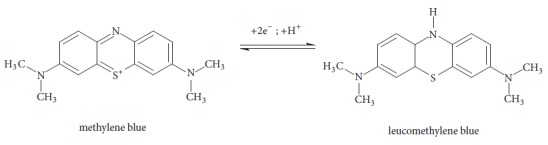
(2)


Moreover, methylene blue is treated as radical's indicator or interceptor for many years, for example, as a retarder of free-radical polymerization of acrylonitrile, methyl methacrylate, and styrene [18] or for rapid detection of hydroxyl radicals [19, 20]. In addition, methylene blue was used as a sensor to determine the total antioxidant potential (CPA) by photometric method, with respect to the hydroxyl radicals generated in the Fenton reaction [21]. Methylene blue was also used to analyse the interaction between it and DNA as a redox indicator of a hybridization event. Variation of the electrochemical signal from the DNA-methylene blue complex was shown in the DNA sequence used [17]. In addition, methylene blue was used as a sensor to determine the total antioxidant potential (CPA) by photometric method, with respect to the hydroxyl radicals generated in the Fenton reaction [21], as well as the design of a novel and efficient electro-Fenton cathode with skeleton of melamine foams [22].

Likewise, degradation mechanism of methylene blue through graphene oxide has been investigated. To enhance the catalytic and separation properties of akaganeite nanoparticles, akaganeite impregnated graphene oxide nanocomposite was fabricated through facile hydrolysis. The degradation intermediates of methylene blue adsorbed on the solid surface of graphene oxide were comprehensively identified with time of flight-secondary ion mass spectroscopy (TOF-SIMS) for the first time in paper [23]. Novel hybrid nanostructures or nanocomposites are becoming more and more important to their newly evolved properties. Alpha-iron(III) oxide anchored to graphene oxide (GO) nanosheet (alpha-Fe_2_O_3_@GO) was synthesised through a facile hydrolysis process and its photo-catalytic durability and performances in heterogeneous Fenton system were evaluated. The decolourisation rates of methylene blue in alpha-Fe_2_O_3_@GO + hydrogen peroxide + UV system within a wide pH range were approximately 3 times higher compared with classical systems. This enhanced decolourisation of methylene blue in new alpha-Fe_2_O_3_@GO + hydrogen peroxide + UV system was attributed to the unique incorporation of graphene peroxide into the catalyst. High efficiencies of degradation were achieved on various organic pollutants (around 96-100%), such as cationic compounds of methylene blue and rhodamine B, anionic compound Orange II and Orange G, neutral compounds of phenol, 2-nitrophenol, and endocrine disrupting compound 17-beta-estradiol. The dominant reactive oxygen species responsible for decolourisation, such as hydroxyl radicals and superoxide anion radicals, generated by activation of hydrogen peroxide on the surface of alpha-Fe_2_O_3_@GO were detected and quantified by free-radical quenching methods. The results lay a foundation for highly effective and durable photo-Fenton technologies for organic wastewater within wider pH ranges than the conventional photo-Fenton reaction [24].

Another research with methylene blue investigates catalyst efficiency for removing dyes from municipal sewage sludge through a nonuniform Fenton reaction. The catalyst, obtained on the basis of seven-water iron sulphate, was used to degrade methylene blue in an aqueous solution. The influence of initial solution pH, catalyst dosage, hydrogen peroxide, and temperature on methylene blue degradation was investigated. The methylene blue removal mechanism was examined. The results showed that methylene blue was able to reach 98% under optimal conditions. The methylene blue removal mechanism was free-radical reaction, in which hydroxyl radicals were generated between hydrogen peroxide and iron sulphate through the heterogeneous Fenton reaction [25]. Another article about a combination of phosphorus oxoanions which was used to enhance the removal efficiency of dyes; methylene blue from wastewater has to be mentioned. The submerged plasma irradiation (SPI) system showed synergistic methylene blue removal efficiency, due to the plasma irradiation and Fenton-like oxidation. When the ferrous ions are released from the iron electrode in the SPI system under plasmonic conditions form complexes with the phosphorus oxoanions anions, they can react with dissolved oxygen or hydrogen peroxide via Fenton-like reactions. The experimental results revealed that a sodium triphosphate combined SPI system has a higher dye removal efficiency than a tetrasodium pyrophosphate or a sodium hexametaphosphate combined SPI system under similar dissolved iron ion concentrations. In addition, it was proved that this system could be used to eliminate six different commercial dyes. The results of this study indicated that the sodium triphosphate/SPI/oxygen system is a promising advanced oxidation approach for dye wastewater treatment [26].

Homogeneous Fenton catalysts in methylene blue degradation were the topic of another article, where to achieve rapid dye removal in oxidation processes, copper ferrite (CuFe_2_O_4_) was isothermally reduced in a hydrogen flow and used as a magnetically separable catalyst for activation of hydrogen peroxide. The physicochemical properties of the reduced copper ferrite were characterized with several techniques, including transmission electron microscopy, X-ray diffraction, X-ray photoelectron spectroscopy, and magnetometry. In the catalytic experiments reduced copper ferrite showed superior catalytic activity compared to raw copper ferrite for the removal of methylene blue due to its relatively high surface area. A limited amount of metal ions was leached from the reduced copper ferrite and these leached ions could act as homogeneous Fenton catalysts in methylene blue degradation. The effects of experimental parameters such as pH, catalyst dosage, and hydrogen peroxide concentration were investigated. Free-radical inhibition experiments and electron spin resonance (ESR) spectroscopy revealed that the main reactive species was hydroxyl radical [27].

The aim of this work was to examine the possibility of using methylene blue as a detector to determine the changes in the concentration of radicals during the gelation process of unsaturated polyester resins. Methylene blue was used for the first time as an indicator of changes in the concentration of these radicals by using its colour change from the colour form to a colourless form (leuko). The tests were carried out for the wavelength of electromagnetic radiation in the UV-Vis spectrum.

## 2. Materials and Methods

The process of radicals creating in unsaturated polyester resins during the gelation process can be monitored by spectroscopic methods using methylene blue, which is a good radical indicator. However, controlling the rate of this reaction in the styrene solution in which cross-linking occurs does not seem to be easy. That is why an attempt was made to use an inert solvent (acetone), which is characterized by much lower density and viscosity compared to unsaturated polyester resin (polyester solution in styrene). Nevertheless, radicals generating system remains unchanged.

In the acetone medium investigated amino modifier, cobalt(II) 2-ethylhexanoate (as accelerator), methylene blue, and butan-2-one peroxide (initiator), respectively, were placed. Standard laboratory conditions were applied. Investigated amino modifiers are as follows:

- 2,2'-((4-chlorophenyl)imino)diethanol,
- 2,2'-((4-bromophenyl)imino)diethanol,
- 2,2'-((4-iodophenyl)imino)diethanol,
- 1,1'-((4-chlorophenyl)imino)dipropan-2-ol,
- 1,1'-((4-bromophenyl)imino)dipropan-2-ol,
- 1,1'-((4-iodophenyl)imino)dipropan-2-ol.

With the use of Thermo Evolution 301 UV-Vis spectrophotometer, the change of absorbance band intensity for the electron transition in the function of the amount of modifier was determined, likewise in the function of the amount of methylene blue as indicator. Each of the modifiers given above was tested for eleven concentrations (0.006, 0.011, 0.023, 0.047, 0.094, 0.188, 0.376, 0.750, 1.500, 3.800, and 7.500 cm^3^ of 0.01 M amine solution). The measurement was carried out in the wavelength range from 400 to 500 nm, with 12 s duration of one pass. Each sample was subjected to 3+5 measurements: three as blind sample, i.e., without initiator (to determine accuracy and precision) and five measurements with 2-butanone peroxide to see absorbance band changes in time. All the measurements were done in quartz cuvettes rinsed with acetone after each test.

Raw data from spectrophotometer is in the form of transmittance as a function of wavelength; therefore the first step of analysis was to transform those data into absorbance form, according to the formula:

(3) $$\begin{array}{c} A=-\log\left(\;\frac{\%T}{100}\;\right) \end{array}$$

where* A* is absorbance;* T* is transmittance.

Transformation is necessary because absorbance is in a logarithm scale and it enables detecting weak signals next to very strong signals. In the case of quantitative analysis, absorbance is simply more suitable.

**Figure 1:**
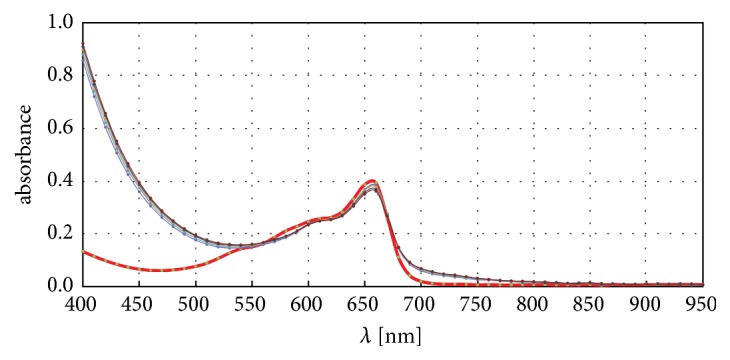
Absorbance in function of wavelength for selected modifier (red thick curves, blind samples).

Effect of that kind of transform is shown on Figure 1, where red thick curves are blind samples (without initiator) while curves with normal thickness and different colours show the changes of absorbance band intensity over time.

Calculations based on statistical methods in experimental sciences [28] were done using STATISTICA 12 application [29]. Modules for statistical parameter calculations and regression analyses were used.

## 3. Results and Discussion

**Figure 2:**
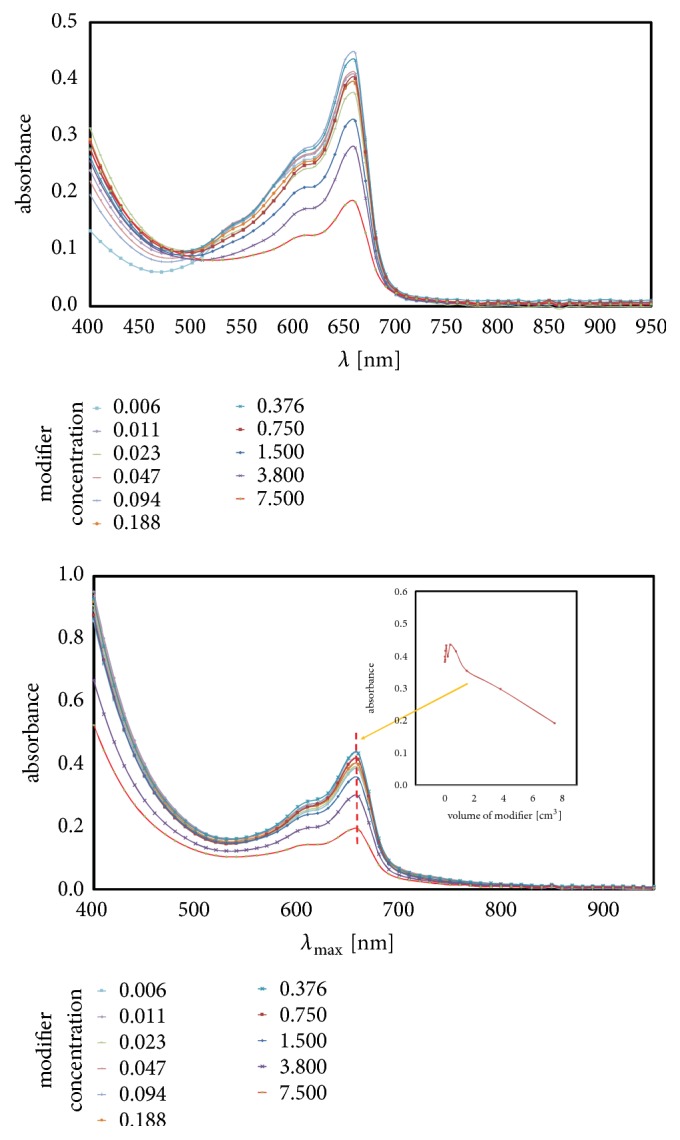
Absorbance band intensity changes in function of eleven concentration values for blind samples (upper) and for eleven concentrations first measurement (bottom).

Blind samples curves taken from all of eleven concentrations in all investigated amino modifiers show some regularity (Figure 2).

**Figure 3:**
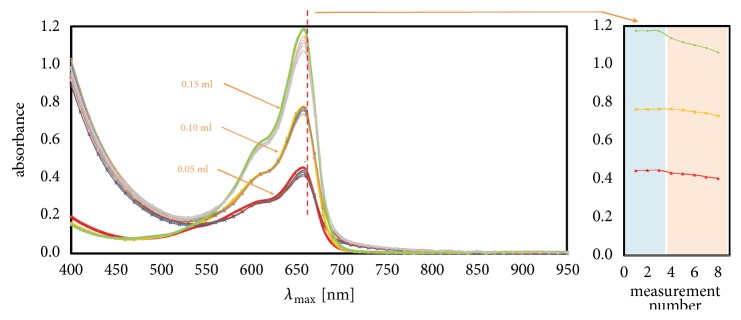
Absorbance curves for selected concentration in function of amount of dye.

For example, between 600 and 700 nm, increase in radicals' activity corresponds to increase in absorbance. Moreover, regularities happen not only for blind samples but for all concentrations. The dependency type (linear, exponential, etc.) can be determined just like on the Figure 2 (small picture). However, the question arises: how does the amount of methylene blue influence on shape or intensity of curves. Three quantities of dye were considered, 0.05, 0.10, and 0.15 cm^3^, and results are shown on Figure 3. The more dye was added; higher value of absorbance was observed in mentioned range.

As seen on the right of Figure 3, all blind samples (thick lines: upper green, middle yellow, and bottom red) were measured three times (measurements 1-3 with blue background) because of its stability in time caused by lack of initiator. Next five measurements (no. 4-8, orange background) were singular; high-speed radical reactions preclude multiple measurements in one point.

**Figure 4:**
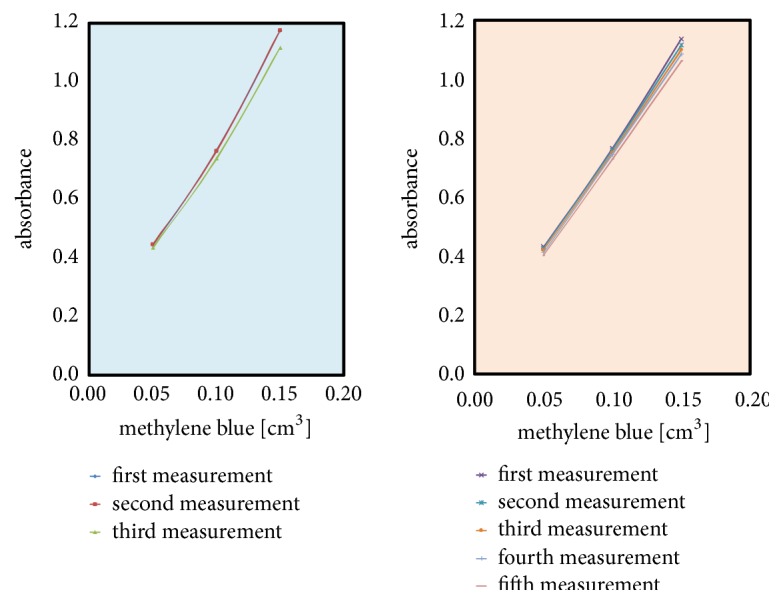
Absorbance at 660 nm for selected concentration in function of amount of dye.

Moreover, in mentioned range 600-700 nm, the maximum of absorbance in all investigated amino modifiers occurs at 660 ± 10 nm (Figure 3). As seen on Figure 4, where on the left (with blue background) are values for blind samples and on the right (with orange background) are normal samples, curves constructed of absorbance values at 660 nm [20] in function of amount of dye have linearity shape with R^2^ (variance) circa 0.99. All values of R^2^ of linear fitted curves for all investigated substances are shown in Table 1 while all measured data are shown in Table 2. Moreover, Table 3 contains calculated statistical parameters that confirm statistical significance of the measures.

**Figure 5:**
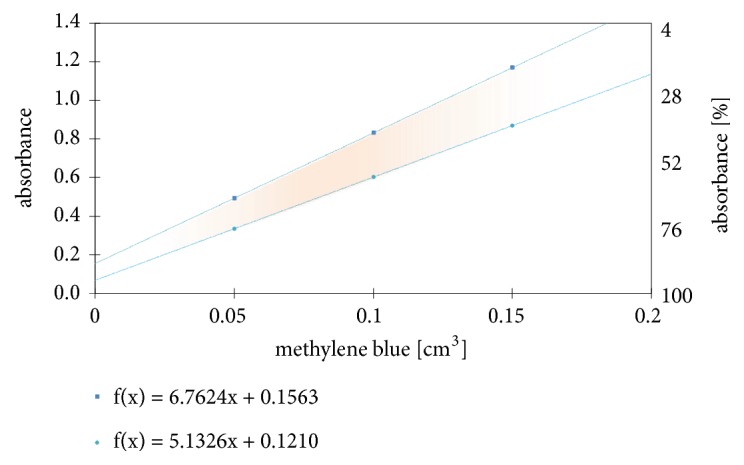
Range of the applicability of the method of measuring the concentration of radicals.

Between the boundary lines presented on Figure 5 the area that contains measuring points from Table 2 is located. It is easy to notice how methylene blue volume affects the absorbance. Moreover, with this graph, it is knowable how to control the range of responses. Too little or too much dye volume does not allow for proper measurement: it is too close to 0 or 100 % of absorbance. Figure 5 shows that best dye volume for such experiments is about 0.1 cm^3^: less than 0.05 cm^3^ gives high value of absorbance and sometimes it can exceed 100 %; however more than 0.15 cm^3^ (absorbance<30%) may cause problems of distinguishing signal from the background.

Boundary lines suggest that, for the lack of methylene blue, absorbance has values in 0.0-0.2 range, but it is misleading. No added dye makes it impossible to study such a system, which is based on the trapping of radicals by the dye molecules. Considering a system in which there is at least one dye molecule, the left vertical axis could be described as an asymptote.

## 4. Conclusions

This paper describes the new methodology that allows analysing radical activity in gelation unsaturated polyester resins reactions. The developed method gives the possibility of measuring the concentration of radicals formed during the gelation process without resorting to expensive and complicated EPR method, requiring immediate freezing of samples during the curing process of unsaturated polyester resin.

The results obtained in this work allow us to state that, in radical polymerization reaction with built-in preaccelerator, the dye amount affects absorbance value in linear way. Observed dependency occurs for

- blind samples measurements (experiments without initiator),
- proper measurements (with time-depend reactions after adding butan-2-one peroxide).

Moreover, linearity mentioned above exists also irrespective of kind of

- substituent at the nitrogen atom (2-hydroxyethyl or 2-hydroxypropyl),
- halogen atom (Cl, Br, I) in para position of aromatic ring.

Analytically, the method of measuring transmittance changes in the UV-Vis spectrum is much more available and cheaper. These measurements can be used to study other polymerization reactions, where the cross-linking process takes place with the participation of radicals.

## Figures and Tables

**Table 1:** Values of R^2^ for linear fitted curves.

Name of modifier	R^2^ (blind)	R^2^ (proper)
2,2'-((4-chlorophenyl)imino)diethanol	0.9951	0.9997
2,2'-((4-bromophenyl)imino)diethanol	0.9271	0.9501
2,2'-((4-iodophenyl)imino)diethanol	0.9999	0.9988
1,1'-((4-chlorophenyl)imino)dipropan-2-ol	0.9918	0.9941
1,1'-((4-bromophenyl)imino)dipropan-2-ol	0.9840	0.9918
1,1'-((4-iodophenyl)imino)dipropan-2-ol	0.9984	0.9997

**Table 2:** Measured data (absorbance values) for blind (1-3 [italic]), average of blind (4 [bold italic]), and proper (5-9) samples in function of methylene blue volume (0.05, 0.10, and 0.15 cm^3^) for six modifiers.

Name	No.	Methylene blue [cm^3^]			Name	No.	Methylene blue [cm^3^]		
		0.05	0.10	0.15			0.05	0.10	0.15
2,2'-((4-chlorophenyl)-imino)diethanol	1	*0.4433*	*0.7649*	*1.1749*	1,1'-((4-chlorophenyl)-imino)dipropan-2-ol	1	*0.4142*	*0.8235*	*1.1204*
	2	*0.4455*	*0.7634*	*1.1747*		2	*0.4143*	*0.8242*	*1.1224*
	3	*0.4455*	*0.7667*	*1.1729*		3	*0.4134*	*0.8224*	*1.1208*
	4	***0.4448***	***0.7650***	***1.1742***		4	***0.4140***	***0.8234***	***1.1212***
	5	0.4320	0.7651	1.1363		5	0.4111	0.7860	1.0789
	6	0.4275	0.7599	1.1160		6	0.4049	0.7766	1.0696
	7	0.4210	0.7504	1.0999		7	0.3968	0.7688	1.0517
	8	0.4117	0.7445	1.0852		8	0.3870	0.7550	1.0282
	9	0.4028	0.7287	1.0614		9	0.3778	0.7379	1.0089

2,2'-((4-bromophenyl)-imino)diethanol	1	*0.3890*	*0.8144*	*0.9640*	1,1'-((4-bromophenyl)-imino)dipropan-2-ol	1	*0.4978*	*0.7577*	*1.1641*
	2	*0.3908*	*0.8156*	*0.9647*		2	*0.4982*	*0.7586*	*1.1678*
	3	*0.3913*	*0.8186*	*0.9622*		3	*0.4986*	*0.7603*	*1.1705*
	4	***0.3904***	***0.8162***	***0.9636***		4	***0.4982***	***0.7589***	***1.1675***
	5	0.4192	0.7861	0.9454		5	0.4507	0.7485	1.1500
	6	0.4192	0.7808	0.9420		6	0.4491	0.7439	1.1441
	7	0.4141	0.7750	0.9291		7	0.4459	0.7375	1.1393
	8	0.4065	0.7648	0.9178		8	0.4410	0.7285	1.1260
	9	0.3990	0.7549	0.9054		9	0.4354	0.7205	1.1199

2,2'-((4-iodophenyl)imino)-diethanol	1	*0.3871*	*0.6618*	*0.9431*	1,1'-((4-iodophenyl)imino)-dipropan-2-ol	1	*0.3922*	*0.7217*	*1.1020*
	2	*0.3905*	*0.6618*	*0.9450*		2	*0.3903*	*0.7220*	*1.1002*
	3	*0.3925*	*0.6643*	*0.9477*		3	*0.3890*	*0.7206*	*1.1018*
	4	***0.3900***	***0.6627***	***0.9452***		4	***0.3905***	***0.7214***	***1.1013***
	5	0.3959	0.6427	0.9157		5	0.3603	0.7105	1.0240
	6	0.3913	0.6354	0.9085		6	0.3585	0.7005	1.0124
	7	0.3839	0.6262	0.8980		7	0.3533	0.6878	1.0032
	8	0.3761	0.6162	0.8854		8	0.3464	0.6710	0.9815
	9	0.3662	0.6003	0.8720		9	0.3381	0.6531	0.9656

**Table 3:** Statistical parameters calculated using Student's t-distribution.

Modifier name	Average difference	Standard deviation of differences	t_calc_	t < t_0.05,2_	p	p>0.05
2,2'-((4-chlorophenyl)-imino)diethanol	0.038474	0.031534	2.1133	YES	0.169	YES
2,2'-((4-bromophenyl)-imino)diethanol	0.019449	0.035484	0.9494	YES	0.443	YES
2,2'-((4-iodophenyl)-imino)diethanol	0.031712	0.021792	2.5205	YES	0.128	YES
1,1'-((4-chlorophenyl)-imino)dipropan-2-ol	0.050234	0.028553	3.0473	YES	0.093	YES
1,1'-((4-bromophenyl)-imino)dipropan-2-ol	0.036154	0.015847	3.9515	YES	0.058	YES
1,1'-((4-iodophenyl)-imino)dipropan-2-ol	0.059986	0.038111	2.7262	YES	0.112	YES

## Data Availability

No data were used to support this study.
